# Post-Laparoendoscopic Single-Site Donor Nephrectomy Ipsilateral Testicular Pain, Does Operative Technique Matter? A Single Center Experience and Review of Literature

**DOI:** 10.1155/2022/3292048

**Published:** 2022-03-23

**Authors:** Hany M. El Hennawy, Abdullah S. Al Faifi, Eisa Al Atta, Omar Safar, Saad Thamer, Weam El Nazer, Ahmed I. Kamal, Abdelaziz A. Abdelaziz, Shaher A. Kawasmeh, Naveed Mirza, Mohammad F. Zaitoun, Khalid Al-Alsheikh, Osama Shalkamy, Ahmed Mahedy

**Affiliations:** ^1^Surgery Department, Section of Transplantation, Armed Forces Hospitals Southern Region, Khamis Mushayte 101, Saudi Arabia; ^2^Urology Department, Armed Forces Hospitals Southern Region, Khamis Mushayte 101, Saudi Arabia; ^3^Mansoura Urology and Nephrology Center, Mansoura University, Mansoura, Egypt; ^4^Pharmacy Department, Armed Forces Hospitals Southern Region, Khamis Mushayte 101, Saudi Arabia; ^5^Urology Department, Faculty of Medicine, Al Azhar University, Cairo, Egypt

## Abstract

**Aim:**

To assess incidence and characteristics of post-laparoendoscopic single-site donor nephrectomy (LESS DN) testicular pain.

**Materials and Methods:**

A prospective comparative study of all male donors post-left LESS DN (group A) vs. postopen nephrectomies (group B) was performed at our center. Patients' demographics, perioperative data, and postoperative consultation reports were reviewed. Testicular pain, swelling, numbness, urinary symptoms, and sexual dysfunction were evaluated. Patients with a history of scrotal pathology or surgical procedure were excluded. Pain and tenderness were scored on a standard 10-point scale.

**Results:**

From September 2017 to December 2020, 85 and 35 male patients of groups A and B met the evaluation criteria. Ipsilateral testicular pain developed in 11 patients (15.3%) and 2 patients (9.5%) in groups A and B, respectively. In most instances, the pain was mild to moderate in severity, started after 6 ± 2.1 and 4 ± 1.1 days postoperatively in groups A and B, respectively. Six patients in group A were evaluated with transscrotal ultrasonography that showed no abnormalities. All patients in both groups responded well to medical treatment.

**Conclusions:**

Post-LESS DN ipsilateral testicular pain is usually mild and self-limited. Preoperative patient education and discussion of the possibility of development of testicular pain and its management should be an integral component of laparoscopic donor nephrectomy informed consent.

## 1. Introduction

Laparoscopic donor nephrectomy (LDN) is currently considered a well-established kidney procurement technique. The main reasons for that are the rate of open nephrectomy (ODN) major complications ranging from 1% to 5%, a higher rate of minor complications from 13% to 30%, and a mortality rate of 0.03% [1]. Generally, pneumothorax, bleeding, and wound care issues are the most prevalent complications with open nephrectomy [1–5]. Despite being relatively safer, previous studies highlighted the association of LDN with ipsilateral testicular pain [2, 6, 7], with an incidence ranging from 8% to 44% of donors [6, 8, 9].

Gjertson CK et al. described the post-LDN testicular pain as mild, self-limited, and often emerged with direct questioning; this finding seemed consistent and recurrent [7]. There are three possible explanations of post-LDN testicular pain: vascular congestion resulting from gonadal vein ligation, testicular ischemia due to testicular artery disruption, and neural damage of the spermatic plexus. It has been proposed that the gonadal vein ligation above the bifurcation of iliac vessels could reduce this complication incidence [8–10]. Few studies compared the incidence of testicular pain in donors undergoing ODN versus LDN. Therefore, this study aimed to investigate the incidence of testicular pain and swelling following laparoendoscopic single-site LESS DN and ODN.

## 2. Methods

A single-center prospective comparative study was conducted, including male patients who underwent left LESS DN (group A) and ODN (group B) performed between Sept 2017 and Oct 2020 at Armed Forces Hospital Southern Region, Khamis Mushayt, to assess postoperative orchialgia. All patients consented to participate in the study. Patients' demographics and perioperative data were collected. The local institutional ethical committee approved the study. Patients with a history of scrotal pathology, testicular pain, or surgical procedure were excluded.

### 2.1. Surgical Procedure

A full detailed description of our LESS DN and ODN operative techniques was mentioned in our previous article [11]. The ODN technique was a standard retroperitoneal flank approach without rib resection. The same surgeons conducted all LESS DN and ODN operations. All surgeries were led by a highly experienced laparoscopic surgeon, with no learning curve necessary. The highest intraabdominal pressure measured was 12–15 mmHg. To avoid ureteral ischemia, our laparoscopic technique entails meticulous preservation of the periureteral tissue while conducting the ureteral dissection down to the common iliac vein.

In ODN, the gonadal vein was ligated distally at the junction with the renal vein. Additionally, the proximal division of the gonadal arteries (using the Ligasure^TM^ Maryland jaw device from Medtronic) and the ureter (with surgical endoclips) was conducted above their junction with the iliac vessels. Between days 3 and 5, patients were discharged with the same analgesic medication.

For patient selection for open donor nephrectomy, patient preference, BMI above 32, and early bifurcation of renal arteries in cases, we prefer an arterial stump and one arterial anastomosis in recipients with bad vessels.

### 2.2. Postoperative Evaluation of Symptoms

On each clinic visit at 1 week and then 2, 3, 6, 8, and 12 months after surgery, assessed postoperative orchialgia (onset, course, duration, and management), testicular swelling, numbness, urinary symptoms, and sexual dysfunction. As recommended by the National Institute of Health (NIH), the standard 10-point numeric rating scale assesses postoperative pain. Zero indicates a complete absence of pain, and ten is severe. Complete relief of pain was considered when the score reached zero.

### 2.3. Statistical Analysis

Data were entered into an SPSS 27.0 spreadsheet (SPSS Inc., Chicago, IL). To compare categorical and continuous variables, we utilized chi-squared and Student's *t*-tests, respectively. The statistical significance level was set at *p* ≤ 0.05, and all *p* values presented were two-sided. Continuous data were described as means and standard deviations, whereas categorical data were presented as proportions and percentages.

## 3. Results

During the study period, a total of 85 male patients post-LESS DN (group A) and 21 post-ODN (group B) met the study inclusion criteria. Patients' characteristics and perioperative data are given in Table 1. Proximal division of the gonadal vein was performed in all LESS DN cases and distal division of gonadal vein in its junction with the left renal vein in all ODN cases.

Ipsilateral testicular pain developed in 11 patients (15.3%) and 2 patients (9.5%) in groups A and B (*P* value ≤0.250), respectively. In most instances, the pain was immediate onset, started after 6 ± 2.1 and 4 ± 1.1 days postoperatively in groups A and B, respectively. The pain was mild to moderate in severity in 82%, and 100% of patients with the mean visual analogue scale score was 4.7 ± 2.1 and 2.1 ± 1.2 in groups A and B. Moreover, the pain subsided after 3 ± 1.1 and 1 ± 0.6 months in groups A and B. Six patients (54.5% of orchialgia group) were evaluated with transscrotal ultrasonography that showed no abnormalities. 73% and 100% of patients responded to paracetamol in groups A and B, respectively. Ipsilateral scrotal swelling was found in groups A and B in 3 patients (4.2%) and 1 patient (4.7%), respectively. Fortunately, all swellings subsided completely with conservative management. In group A, ipsilateral scrotal and thigh numbness, pain during intercourse, and the number of medical consultations for testicular symptoms are observed in 4, 3, and 6 patients.  Table 2 provides a detailed comparison between patients with and without orchialgia post-LESS DN. The orchialgia group was older, received less intravenous fluid intraoperatively, produced less urine intraoperatively, and had a shorter hospital stay than the nonorchialgia group of patients.

## 4. Discussion

The incidence of post-LDN ipsilateral testicular discomfort ranges from 1% of 381 patients [12], 3% of 70 patients [13], 2.2% of 20 patients [14], 6.2% of 129 patients [15], 8.5% of 440 patients [8], 9.6% of 145 patients [2], 26.1% of 20 patients [9], and 50% of 20 patients [7]. In this cohort, 15% of our LESS DN patients had ipsilateral testicular pain. An overview of studies on post-LDN ipsilateral testicular pain is given in Table 3.

The mechanism of postnephrectomy testicular pain is still uncertain. Several researchers [2, 6, 8, 16] proposed that it has been caused by damage to nerve fibers of the spermatic plexus during gonadal vein and ureter cutting. Furthermore, gonadal vein ligation has been linked to testicular vascular congestion and pain [7]. Additionally, it has been proposed that orchialgia and hydrocele are caused by an interruption in lymphatic drainage [7, 9, 17]. Sureka et al. suggested that the probability of orchialgia would be increased if the surrounding fatty tissue (which may contain neural fibers) was entrapped during gonadal vein and ureter clipping [10].

Postoperative pain is often underestimated [18], as lesser than half of patients (41.7%) suffering from testicular symptoms sought medical advice [9]. Similarly, 8.3% of post-LESS DN patients in this study population sought medical advice. Several studies reported that testicular pain develops after hospital discharge (within 4 to 7 days following surgery). However, it is often minor and self-limiting, making it easily overlooked by the clinicians unless mainly investigated [3, 4, 7].

The pain mostly subsided within three months of surgery, and 73% of cases responded to paracetamol alone. Similarly, 83% of group A patients had mild-to-severe pain that began approximately a week postoperatively in our research. Furthermore, it lasted for a few days to several weeks and was associated with testicular swelling in 10 of 11 cases [6], and the pain lasted for a mean of 13–4.2 (range 4–30 days) days and improved with conservative management [10].

However, the pain may impede the return to usual daily activities and, as a result, have a significant influence on the quality of life [6]. Fortunately, neither group's participants had testicular pain or swelling that interfered with their professional or daily lives.

Patients with post-LDN testicular discomfort should have a medical evaluation. Doppler ultrasonography may be performed based on pain characteristics and a physical examination [9]. Only six individuals in our study underwent transscrotal ultrasonography, which revealed no abnormalities.

Patients with idiopathic orchialgia are generally treated medically with analgesics, nonsteroidal anti-inflammatory medications, and antidepressants [19]. As per Pinar et al., more than 70% of patients controlled their pain with no more than nonopioid analgesic (paracetamol), with a mean duration of 15 months [9]. If medical therapy fails, surgical treatment may be indicated, including inguinal orchiectomy, with 73% of patients experiencing pain relief [20], laparoscopic cord denervation at the level of the inguinal ring in patients who respond to cord block [21], and neurectomy of the ilioinguinal or genitofemoral nerve proximal to the site of neuroma or entrapment [22].

Pinar et al. suggested that patients with atypical or long-lasting testicular pain (as determined by routine clinical examination and Doppler ultrasonography) should be referred to chronic pain management specialists to assess neuropathic pain and provide appropriate treatment [9].

To prevent postoperative testicular pain after laparoscopic hand-assisted nephrectomy, several researchers proposed preserving the gonadal vein and ligating it at the confluence of the renal vein [2, 15]. Sureka et al. reported in 2015 that ligating the gonadal vein above the iliac vessels dramatically decreased orchialgia, 2.5% versus 30% if ligated below the iliac vessels [10]. Furthermore, Ugo Pinar et al. proposed avoiding tissue incision around the gonadal vein to minimize neural fiber damage [9]. Burgos et al. indicated that appropriate perioperative intravascular volume expansion could prevent a drop in renal blood flow during LDN, which may decrease the likelihood of orchialgia postoperatively [23].

It is necessary to provide preoperative patient education and complete informed consent to define the risk of postoperative testicular events. Furthermore, patients should be adequately counseled postoperatively to manage mild pain with nonopioid analgesic (if required) and consult if they suffer unusual pain, such as sudden, severe, or long-lasting pain, or if analgesic failed [9].

### 4.1. Limitation of the Study

The main limitation of this study is the small imbalanced sample size of patients. Moreover, detection bias and overestimation of complaints rate could not be excluded. However, we recruited all patients who met the study inclusion criteria.

## 5. Conclusions

Post-LESS DN ipsilateral testicular pain was generally mild and self-limited. However, patients should be informed about orchialgia risk during the predonation medical discussion.

## Figures and Tables

**Table 1:** Patients' characteristics and perioperative data.

Mean (±SD)	LESS DN	ODN	*P* value
	*N* = 85	*N* = 21	
Age (years)	30.1 ± 10.4	29.7 ± 14	≤0.452
BMI	24.8 ± 4.5	28.1 ± 2.1	≤0.0015
Vascular anatomy			
Single artery	68	28	—
Two arteries	17	7	—
Retroaortic vein	4	1	—
Early bifurcation of artery	0	5	—
Operative time	175.9 ± 24.9	130 ± 22.7	≤0.0001
WIT (min)	5.2 ± 1.02	2.1 ± 1.0	≤0.0001
IVF (liters)	3.3 ± 0.56	2.9 ± 1.1	≤0.061
UOP (ml)	750 ± 26.6	640 ± 32.3	≤0.0001
EBL (ml)	90 ± 20	70 ± 25.5	≤0.001
LOS (days)	3.5 ± 1.4	2.9 ± 1.1	≤0.024
Follow-up (months)	8 ± 2.3	6 ± 3.4	≤0.009
Testicular pain			
Ipsilateral	11 (15.3%)	2 (9.5%)	≤0.250
Contralateral	0	0	—
Bilateral	0	0	—
Pain intensity			
Mild	8 (72.7%)	2 (100%)	≤0.004
Moderate	1 (9%)	—	—
Severe	2 (18.3%)	—	—
Visual analogue scale score	4.7 ± 2.1	2.1 ± 1.2	≤0.0001
Pain			
Started (days postoperatively)	6 ± 2.1	4 ± 1.1	≤0.0001
Subsided (months postoperatively)	3 ± 1.1	1 ± 0.6	≤0.0001
Ipsilateral scrotal swelling	3 (4.2%)	1 (4.7%)	≤0.460
Ipsilateral scrotal and thigh numbness	4 (5.5%)	0	—
Pain during intercourse	3 (4.2%)	0	—
Number of combined testicular tenderness and hydrocele	3 (4.2%)	0	—
Urinary symptoms	0	0	—
Sexual dysfunction	0	0	—
Number of medical consultation for testicular symptoms	6 (8.3%)	0	—
Workup (scrotal USG)	6 (8.3%)	0	—
Treatment			
Paracetamol	8	2	—
NSAID	4	0	—
Antidepressant	2	0	—
Surgery	0	0	—

WIT, warm ischemia time; IVF, intravenous fluid; UOP, urine output; EBL, estimated blood loss; LOS, length of hospital stay; USG, ultrasonography.

**Table 2:** Orchialgia vs. nonorchialgia in LESS DN patients.

Mean (±SD)	Orchialgia group	Nonorchialgia group	*P* value
Number	11	61	—
Age (years)	35.7 ± 5.2	31.2 ± 4.4	≤0.011
Operative time (mins)	175.6 ± 24.9	166.6 ± 9.9	≤0.138
EBL (ml)	87 ± 27	90 ± 20	≤0.365
IVF (liters)	3.3 ± 0.56	4.3 ± 0.62	≤0.0001
UOP (ml)	650 ± 26.6	750 ± 20.6	≤0.0001
WIT (min)	4.8 ± 1.02	5.2 ± 1.02	≤0.133
LOS (days)	2.8 ± 1.1	3.5 ± 1.4	≤0.042
Testicular antecedents	No	No	—
Contralateral orchialgia	No	No	—
Bilateral orchialgia	No	No	—

WIT, warm ischemia time; IVF, intravenous fluid; UOP, urine output; EBL, estimated blood loss; LOS, length of hospital stay; CIV, common iliac vein.

**Table 3:** Summary of studies of postlaparoscopic donor nephrectomy testicular pain.

Study, year	Study type	Study size	Ipsilateral testicular pain, %	Testicular pain characteristics	Notes
Kim et al., 2003 [2]	Retrospective	145	9.6	Onset: between D1–D14 with a mean of 5.4 ± 4.5 days	One patient had no testicular flow, negative exploration
				Resolved: ranged from 2 weeks to 22 months with a mean of 6.3 ± 7.2 months	One patient underwent ipsilateral spermatocelectomy, 86% responded to conservative management
Gjertson Sundaram, 2008 [7]	prospective	64	Total: 21	Mean intensity was 4 (range 1–8)	—
			When gonadal vein ligated: 33	Resolved: median of 34 days after surgery (range 7–110)	
			Preserved: 3.4		
Jalali et al., 2012 [6]	Prospective	LDN:25	LDN group: 44	Onset: immediately after the operation and lasting for up to 4 weeks	LDN: one scrotal swelling
		ODN: 25	ODN group: 8	Mean intensity was 4.6 (SD: 2.0)	ODN: 100% with mild pain resolved within 2 weeks of surgery
Sureka et al., 2015 [10]	Prospective ligation of the ureter and GV	Group A: 70	Group A: 14.4	Mean intensity was: 3.1 (range 2–5)	All of them had pain relief with conservative management by 13 ± 4.2 days (range 4–30 days).
	Group A: 40 pts above 30 pts below CIV level	Group B: 45	Group B: 0	Onset: in the first week (range 2–7, mean 3.2 ± 0.6 days)	
	Group B: all above the CIV level				
Pinar et al., 2021 [9]	Retrospective phone questionnaire	69	26.1	Onset: 1 month (0.25–6)	16% had scrotal swelling and 3% were hydrocele
				Duration: 15.5 months (1–36)	Medical consultation: 41.7%
					No treatment: 36.4%
					Paracetamol: 36.4%
					NSAIDs: 13.6%
					Amitriptyline: 4.5%

## Data Availability

No data were used to support this study.

## References

[1] Philosophe B., Kuo P. C., Schweitzer E. J. (1999). Laparoscopic versus open donor nephrectomy. *Transplantation*.

[2] Kim F. J., Pinto P., Su L. M. (2003). Ipsilateral orchialgia after laparoscopic donor nephrectomy. *Journal of Endourology*.

[3] Chan D. Y., Fabrizio M. D., Ratner L. E., Kavoussi L. R. (2000). Complications of laparoscopic live donor nephrectomy: the first 175 cases. *Transplantation Proceedings*.

[4] Kim F. J., Ratner L. E., Kavoussi L. R. (2000). Renal transplantation: laparoscopic live donor nephrectomy. *Urologic Clinics of North America*.

[5] Garcia-Covarrubias L., Prieto-Olivares P., Bahena-Portillo A. (2018). Experience and security of the hand-assisted laparoscopic nephrectomy of a living donor in a public health center. *Transplantation Proceedings*.

[6] Jalali, Rahmani S., Joyce A. D., Cartledge J. J., Lewis M. H., Ahmad N. (2012). Laparoscopic donor nephrectomy: an increasingly common cause for testicular pain and swelling. *Annals of the Royal College of Surgeons of England*.

[7] Gjertson C. K., Sundaram C. P. (2008). Testicular pain following laparoscopic renal surgery. *The Journal of Urology*.

[8] Srivastava A., Kapoor R., Srivastava A., Ansari M. S., Singh M., Kapoor R. (2013). Orchialgia after laproscopic renal surgery: a common problem with questionable etiology. Are there any predictors?. *World Journal of Urology*.

[9] Pinar U., Pettenati C., Hurel S. (2021). Persistent orchialgia after laparoscopic living-donor nephrectomy: an underestimated complication requiring information adjustment. *World Journal of Urology*.

[10] Sureka S. K., Srivastava A., Agarwal S. (2015). Prevention of orchialgia after left-sided laparoscopic donor nephrectomy-a prospective study. *Journal of Endourology*.

[11] El Hennawy H. M., Al Hashemy A., Kadi N. M. (2019). Transumbilicallaparoendoscopic single-site donor nephrectomy: evolving trends. *Surgical Endoscopy*.

[12] Su L.-M., Ratner L. E., Montgomery R. A. (2004). Laparoscopic live donor nephrectomy. *Annals of Surgery*.

[13] Brook N. R., Harper S. J., Waller J. R., Nicholson M. L. (2005). A consecutive series of 70 laparoscopic donor nephrectomies demonstrates the safety of this new operation. *Transplantation Proceedings*.

[14] Permpongkosol S., Link R. E., Su L.-M. (2007). Complications of 2,775 urological laparoscopic procedures: 1993 to 2005. *Journal of Urology*.

[15] Shirodkar S. P., Gorin M. A., Sageshima J. (2011). Technical modification for laparoscopic donor nephrectomy to minimize testicular pain: a complication with significant morbidity. *American Journal of Transplantation*.

[16] Starling J. R., Harms B. A. (1989). Diagnosis and treatment of genitofemoral and ilioinguinal neuralgia. *World Journal of Surgery*.

[17] Hassan J. M., Adams M. C., Pope J. C., Demarco R. T., Brock J. W. (2006). Hydrocele formation following laparoscopic varicocelectomy. *Journal of Urology*.

[18] Clemens K. K., Thiessen-Philbrook H., Parikh C. R. (2006). Psychosocial health of living kidney donors: a systematic review. *American Journal of Transplantation*.

[19] Baum N., Defidio L. (1995). Chronic testicular pain: a workup and treatment guide for the primary care physician. *Postgraduate Medical Journal*.

[20] Levine L. A., Matkov T. G. (2001). Microsurgical denervation of the spermatic cord as primary surgical treatment of chronic orchialgia. *The Journal of Urology*.

[21] Cadeddu J. A., Bishoff J. T., Chan D. Y. (1999). Laparoscopic testicular denervation for chronic orchalgia. *The Journal of Urology*.

[22] Kennedy E. M., Harms B. A., Starling J. R. (1994). Absence of maladaptive neuronal plasticity after genitofemoral–ilioinguinal neurectomy. *Surgery*.

[23] Burgos F. J., Saenz J., Correa C. (2009). Changes in visceral flow induced by laparoscopic and open living-donor nephrectomy: experimental model. *Transplantation Proceedings*.

